# Accumulation of Sulforaphane and Alliin in Human Prostate Tissue

**DOI:** 10.3390/nu14163263

**Published:** 2022-08-10

**Authors:** Tracey L. Livingstone, Shikha Saha, Federico Bernuzzi, George M. Savva, Perla Troncoso-Rey, Maria H. Traka, Robert D. Mills, Richard Y. Ball, Richard F. Mithen

**Affiliations:** 1 Quadram Institute Bioscience, Norwich Research Park, Norwich NR4 7UQ, UK; 2 Norfolk and Norwich University Hospital, Colney Lane, Norwich NR4 7UY, UK; 3 Liggins Institute, Waipapa Taumata Rau—University of Auckland, 85 Park Road, Private Bag 92019, Auckland 1142, New Zealand

**Keywords:** *Broccoli*, glucoraphanin, sulforaphane, prostate cancer, garlic, alliin

## Abstract

Diets rich in cruciferous vegetables have been associated with a lower risk of incidence and progression of prostate cancer. Sulforaphane, an isothiocyanate derived from 4-methylsulphinylbutyl glucosinolate (glucoraphanin) that accumulates in certain of these vegetables, notably broccoli, has been implicated in their protective effects. Likewise, the consumption of garlic and its sulphur-containing compounds such as alliin have been associated with a reduction in risk of prostate cancer. In this study, we tested whether consuming glucoraphanin derived from broccoli seeds and alliin derived from garlic resulted in the occurrence of these potential bioactive compounds in the prostate, which may contribute to our understanding of the putative protective effects of these dietary components. We recruited 42 men scheduled for a trans-perineal prostate biopsy into a randomised, double-blinded, 2 × 2-factorial dietary supplement four-week intervention study, and 39 completed the study. The two active interventions were supplements providing glucoraphanin from broccoli (BroccoMax^®^) and alliin from garlic (Kwai Heartcare^®^). Following the intervention, prostate biopsy tissue was analysed for the presence of sulforaphane and its thiol conjugates and for alliin and associated metabolites. Sulforaphane occurred in significantly higher levels in the prostate tissue (both within the transition and peripheral zone) of men consuming the glucoraphanin containing supplements (*p* < 0.0001) compared to men not consuming these supplements. However, while alliin and alliin-derived metabolites were detected within the prostate, there was no significant difference in the concentrations of these compounds in the prostate of men consuming supplements derived from garlic compared to men not consuming these supplements.

## 1. Introduction

Diets rich in cruciferous vegetables such as broccoli have been correlated with a reduction in the risk of aggressive prostate cancer [1,2,3]. The protective activity has been associated with the biological activity of degradation products of glucosinolates that accumulate within these vegetables [4,5,6]. When consumed, glucosinolates are degraded due to the action of plant thioglucosidases (myrosinases) or, if these enzymes have been inactivated by cooking, by microbial activity within the gastrointestinal tract [7]. Foremost amongst the glucosinolates found in broccoli is 4-methylsulphinylbutyl glucosinolate (glucoraphanin, GRN), which is hydrolysed to produce the isothiocyanate sulforaphane (SFN). Within many model cell and animal systems, sulforaphane has been shown to exert biological activity that would be consistent with protection against prostate cancer [4,8,9]. Furthermore, evidence from two human intervention studies indicates that consumption of high-glucoraphanin broccoli either cooked from frozen or made into a broccoli soup suppressed changes in oncogenic gene expression within the prostate tissue of men who were at risk of prostate cancer in a manner that was consistent with a reduction in the risk of aggressive prostate cancer [5,10]. Despite epidemiological and experimental studies, there remain questions concerning the manner by which glucoraphanin and sulforaphane may be able to affect the health of prostate tissue. Pharmacokinetic studies have shown that even after consuming glucoraphanin-enriched broccoli, the level of sulforaphane in the systemic circulation peaks at about 1 μM and is barely detectable after 24 h [11]. Thus, in humans, the exposure of prostate tissue via the systemic circulation is likely to be below that which is commonly used in cell studies in which prostate cells are exposed to concentrations of typically between 1 and 20 μM for up to 72 h [4,12]. Likewise, the levels of sulforaphane that experimental rodents are exposed to either through oral gavage or within chow exceeds the levels to which humans would be exposed from dietary sources.

There are three possible routes by which sulforaphane may affect organs such as the prostate gland. First, sulforaphane may impact hepatic metabolism following first-pass exposure following absorption from the gastrointestinal tract, resulting in induction of NF-E2–related factor 2 (‘Nrf2’)-mediated transcription that can have positive effects on metabolism [13] which, in due, course influences the health of peripheral tissues such as the prostate gland. Second, sulforaphane within the systemic circulation may be sufficient to induce changes in gene expression and metabolism despite the low and transient concentration in which sulforaphane occurs in the circulation following cruciferous vegetable consumption [11]. Third, it is possible that sulforaphane may accumulate within the prostate tissue itself either via the circulation or through urinary reflux and achieve a local concentration sufficient to induce changes that promote prostate health. These changes may include the inhibition of growth of cancerous clones and the reduction of cell oncogenesis. Whilst there is some evidence from rodent studies that sulforaphane can accumulate in the prostate gland [14], there is currently no evidence for accumulation of sulforaphane in the human prostate gland.

Garlic and other *Allium* species have also been associated with the prevention or progression of prostate cancer [15,16,17], and it is possible that sulphur-containing metabolites from these foods may also accumulate in human prostate tissue. The predominant sulphur-containing metabolite in garlic is alliin (S-alkyl-L-cysteine S-oxide), which is odourless and non-volatile. When the plant tissue is damaged, alliinase enzymes rapidly convert alliin to allysulfenates that condense to form allicin (S-allyl prop-2-ene-1-sulfinothionate) and other thiosulfinates, predominantly γ-glutamyl S-allyl-L cysteine (γ-SAC) and S-allyl-L cysteine (SAC) [18].

In this study, we test the hypothesis that consuming glucoraphanin (from broccoli) or alliin (from garlic) results in the accumulation of sulforaphane and alliin and their associated metabolites in the peripheral zone (PZ) and transition zone (TZ) of the human prostate gland. We recruited men scheduled for a trans-perineal prostate biopsy as part of their clinical care into a randomised, double-blinded, 2 × 2-factorial dietary supplement intervention study. Three types of supplements were provided: one that provided glucoraphanin from broccoli (BroccoMax^®^, Jarrow Formulas, Los Angeles, CA, USA), one that provided alliin from garlic (Kwai Heartcare^®^), Kwai, Bury St Edmunds, UK), and a placebo. Urine samples were collected after 24 h. Following a minimum of four-week intervention, biopsy samples from the peripheral and transition zone of the prostate were analysed for the presence of sulforaphane (from the broccoli-derived supplements) and alliin (from the garlic-derived supplements). The short-term nature of the study precludes any assessment of the effect of the supplementation on clinical outcomes. 

## 2. Materials and Methods

### 2.1. Analyses of Glucoraphanin and Sulforaphane

BroccoMax^®^ supplements (Jarrow Formulas, Los Angeles, CA, USA) that contain glucoraphanin derived from broccoli seeds have been used in human trials at doses ranging from 2 to 6 capsules per day with good compliance, tolerability, and no adverse reactions [19,20,21]. To quantify the amount of glucoraphanin within individual capsules, glucoraphanin was extracted from ten capsules from different batches and quantified by LC-DAD-MS as described previously [22]. Briefly, glucosinolates were extracted from the ground content of capsules in 70% (*v*/*v*) aqueous methanol. Then, 50 μL of 16 mM sinigrin were added to 10 mL aliquot of the extract as an internal standard and incubated at 70 °C for 25 min. After cooling to room temperature and centrifugation, 3 mL of liquid supernatant was added to an ion exchange column. Following washing with 0.5 mL water and 0.5 mL 0.02 M sodium acetate buffer (pH 5), 75 μL purified sulfatase (Sigma, St. Louis, MO, USA) was added to the column. The next day, desulphated glucosinolates were eluted from the column with 1.25 mL of Milli-Q^®^ water (Sigma, St. Louis, MO, USA) and analysed by LC-DAD-MS as previously described [22]. Sulforaphane and its thiol conjugates, namely sulforaphane-cysteine (SFN-Cys), sulforaphane-N-acetyl cysteine (SFN-NAC), and erucin-NAC (E-NAC), were analysed in urine and prostate tissues samples as previously described [23] with slight modifications; an Acquity UPLC, HSS T3 1.8 um, 2.1 × 100 mm column was used instead of Phenomenex^®^ Luna 3u C18 (2) 100A 2.1 × 100 mm column (Phenomenex, Torrence, CA, USA) due to improved efficiency for separation and sensitivity, and d8-sulforaphane was used as an internal standard. 

### 2.2. Analyses of Alliin and Associated Metabolites

Kwai Heartcare^®^ supplements (Klosterfrau Healthcare Group, Cologne, Germany) that contain garlic-derived metabolites have previously been used in human clinical studies [24,25] and have been well-tolerated at a dose of 3 tablets/day. Four garlic-derived metabolites were analysed within the supplements: alliin, allicin, γ-SAC, and SAC. For quantification of alliin, 500 mg of each of eight ground supplements were extracted with 25 mL of 20 mM CMA buffer (alliinase inhibitor). Then, 1 mL of extract was centrifuged at 15,000 rpm for 10 min at 4 °C. Alliin was analysed using ion pair HPLC-UV (Luna C18 250 mm × 4.6 mm column) as previously described [26]. γ-glutamyl S-allyl-L cysteine (γ-SAC), S-allyl-L cysteine (SAC), and allicin were analysed using LC-MS/MS as previously described [27]. Analyses of alliin, allicin, γ-SAC, and SAC were undertaken in urine and prostate tissue samples using the same methods.

### 2.3. Preparation of Placebo Capsules

Production of 20,000 placebo capsules was undertaken with the use of the Profiller 1100, in which microcrystalline cellulose powder (MCC) was used to encapsulate size 0 hydroxypropylmethylcellulose capsules. MCC is a purified, partly depolymerised cellulose with shorter, crystalline polymer chains. It has a strong binding performance and is therefore commonly used as a filler and binder in drug formulations. MCC filler was poured and spread evenly to fill all capsules, using a tamper to pack the powder where necessary. The capsules were sieved with granulated sugar to remove any excess MCC from the capsule exterior and visually inspected to ensure quality.

### 2.4. Norfolk ADaPt Study Design

The primary aim of the Norfolk ADaPt study was to test the hypothesis that sulforaphane and alliin from broccoli-derived or garlic-derived dietary supplements accumulate in prostate tissue. The secondary aims were: (1) to determine whether there was differing spatial accumulation of these compounds between the transition and peripheral zones of the prostate; (2) to quantify the level of dietary compounds in urine over a 24-h period following consumption of the supplements; and (3) to assess whether the above endpoints are influenced by the glutathione S-transferase Mu 1 (GSTM1) genotype. The study also sought to test whether dietary supplementation resulted in a difference in human gene expression using next-generation RNA sequencing, and these studies are reported separately [28]

**Figure 1:**
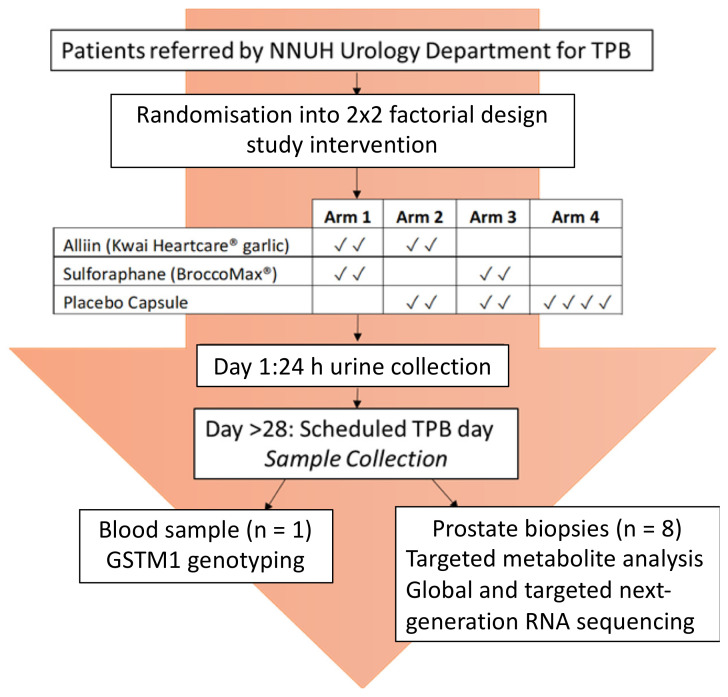
Norfolk ADaPt Study Design.

The study was of a randomised, double-blinded, 2 × 2-factorial design, delivering high-dose dietary supplement interventions to men in the pre-biopsy window prior to their trans-perineal prostate biopsy (TPB) (Figure 1). The study design was chosen since it is effective at increasing the power of a study that involves two treatment arms (glucoraphanin and alliin) and a placebo. It assumes that there is no interaction of the two treatments. 

Forty-two men were recruited, forty of which were randomised in blocks of four to one of the four study arms in which they were asked to consume a pre-determined combination of supplements from two of the three types used in the study: the glucoraphanin supplement (BroccoMax), the garlic or alliin supplement (Kwai), or the placebo supplement (Figure 1). The men were asked to consume two capsules per supplement type, making a total of four capsules once a day for a minimum of four weeks (Figure 1). Participants completed a health questionnaire and two food frequency questionnaires prior to their procedure. On day one of the study following consumption of their first four capsules, participants collected all urine passed for 24 h. On the final day of the study (the scheduled biopsy day), and at least 24 h following consumption of supplements, samples of whole blood, urine, and prostate tissue were collected. The TPB was performed as per their routine clinical care, with eight additional tissue cores taken for research purposes. Follow-up care was continued by the urology consultants at the Norfolk and Norwich University Hospital (NNUH).

The protocol was approved by the Human Research Governance Committee (HRGC QIB 02/2019) of the Quadram Institute Bioscience in February 2019. The East of England—Cambridge East Research Ethics Committee (REC) and the NHS Health Research Authority (HRA) gave full ethical approval in June 2019, and the trial was registered on a publicly accessible database (ClinicalTrials.gov, NCT04046653). 

### 2.5. Study Population and Procedures

Men aged 18–80 with a body mass index (BMI) between 19.5 and 35 kg/m^2^ who were on the waiting list for TPB at the Norfolk and Norwich University Hospital as part of their routine clinical care formed the sampling frame for the study. All men on the waiting list were included regardless of whether they had a confirmed diagnosis of prostatic cancer on active surveillance or were under investigation for suspected prostatic cancer. The inclusion and exclusion criteria are shown in Table 1. If any participants enrolled on the study were offered a biopsy date within the 4-week intervention period, they were excluded from the study to ensure that there was no delay in their routine clinical care. Written consent from participants for undertaking the study and tissue banking of samples was obtained. Participants were randomised into one of four study arms and provided with containers containing the study interventions. Randomisation was performed by a third party with the use of an online randomisation generator. The method of “block randomisation” ensured that participants were evenly distributed between all study arms. Allocation sequence concealment was undertaken and codes placed in an envelope for each participant, ensuring the study scientists were aware of neither the treatment code the patient would receive nor which of the interventions the treatment code corresponded to.

Following consumption of four capsules on day one of the ADaPt study, participants collected all urine passes for 24 h in a 3 L universal container containing 1 g ascorbic acid. Following collection and mixing, the sample was weighed, and urine was divided into 1 mL aliquots and frozen at −80 °C until required for analysis. 

On the final day of the study, participants underwent their scheduled TPB procedure. A standard NHS consent form was completed by the urologist undertaking their TPB procedure as per routine practice. TPB was performed under general or spinal anaesthetic with antibiotic prophylaxis. A blood sample for genotype analysis was taken at the same time as cannulation for anaesthesia, either by the anaesthetist or urologist, to prevent the requirement for further venepuncture. The patient was positioned supine in the lithotomy position (with legs in stirrups). The prostate was visualised via an ultrasound probe placed into the rectum and a template grid with holes spaced at 5 mm intervals positioned against the sterilised skin of the perineum. The prostate gland was then systematically sampled for clinical purposes. For the purpose of the ADaPt study, eight prostate biopsies were taken at the start of the procedure. They were specifically taken from areas not known or suspected to contain cancer. Four cores (two from each of the PZ and TZ) were placed directly into RNAlater solution for next-generation RNA sequencing, three (two from the PZ and one from the TZ) were immediately snap-frozen for targeted metabolite analysis, and one placed directly into 80% methanol. Samples in methanol were incubated at room temperature for 24 h. Tissue samples snap-frozen were immediately placed on dry ice and transferred to the QIB laboratory for storage at −80 °C. All biopsies were carried out by a single consultant urologist (Mr. Robert Mills) at the NNUH to minimise variation in prostate sampling. 

Biopsies taken for clinical purposes (non-study purposes) were fixed in formalin and then processed to paraffin wax blocks using laboratory protocols, and 4 μM haematoxylin and eosin-stained sections were reported (including Gleason grading and attributing a grade group) according to the current best practice. The distribution of neoplastic lesions was delineated in the samples provided for assessment. The material for diagnostic purposes was reported to the clinical team in the standard electronic form. The tissue analysed for research purposes was reported in the same manner as above but relayed to the research team anonymously.

DNA was extracted from whole blood samples using the QIAamp DNA minikit following the manufacturer’s instructions (Qiagen Inc., Hilden, Germany). Both the DNA and 260:280 ratio were quantified on a Nanodrop™ spectrophotometer (ThermoFisher, Waltham, MA, USA). Genotype was determined with 20 ng genomic DNA in a StepOnePlus real-time PCR system (Applied Biosystems, Waltham, MA, USA), with GSTM1 primer (ThermoFisher) and Taqman™ universal master mix II (Applied Biosystems). A 10 min activation period was carried out at 95 °C, followed by 40 PCR cycles at 92 °C for 15 s and 60 °C for 90 s. Allelic discrimination of GSTM1 genotype was determined using StepOne™ v2.3 software (Applied Biosystems).

The Arizona Cruciferous Vegetable Food Frequency Questionnaire’(CVFFQ), developed by the University of Arizona, was completed by participants either prior to or on day of the biopsy to assess cruciferous vegetable intake (as well as other foodstuffs known to be sources of isothiocyanates) during the period of the study. The CVFFQ has previously been shown to provide reproducible, valid estimates of cruciferous vegetable exposure and improved relationships between crucifer consumption and urinary dithiocarbamate, a biomarker of cruciferous vegetable exposure [29]. Data were adjusted according to portion size and cooking method. A non-validated allium FFQ was completed either prior to or on the study day. This FFQ was developed by the study team, in the absence of any validated allium questionnaires, to provide a qualitative indication of allium intake only and was designed primarily to investigate whether there were any individuals with exceptionally high alliaceous vegetable intake. 

### 2.6. Analyses of Dietary Metabolites in Prostate Tissue Samples

Individual snap-frozen prostate biopsy cores (one from each of PZ and TZ per patient) were weighed on a high-sensitivity balance and transferred to screw-top tubes. Then, 200 µL of cold Milli-Q^®^ water and 300 mg of autoclaved, acid-washed 710 to 1180 µm glass beads (Sigma) were added to each tube. The tissue was completely homogenised using a DNA Fast-Prep^®^ (MP Biomedicals, Santa Ana, CA, USA) at 4.0 m/s for 3 cycles of 60 s each. The samples were then placed on a revolving shaker for 15 min at 4 °C. The tubes were centrifuged a 17,000× *g* for 10 min at 4 °C and 50 µL of supernatant transferred to a new Eppendorf. Dietary metabolites were analysed as described above.

### 2.7. Statistical Analysis and Sample Size Determination

While the study was designed as a 2 × 2-factorial study for efficiency, the primary outcomes for each treatment were separate. Thus, the two main comparisons (GRN interventions compared to non-GRN interventions and alliin interventions compared to non-alliin interventions) were analysed independently using Mann–Whitney tests. The primary outcome (total concentration in the prostate) was calculated by averaging the values from the TZ and PZ. 

There were prior data neither on the likely level of accumulation of bioactives in the prostate nor on its variability nor baseline level given usual diet. Moreover, there is no evidence on what levels are likely to be clinically relevant. Hence, a target sample size of 40 was chosen based on pragmatic considerations, leading to an anticipated 20 samples per group for each comparison. 

## 3. Results

### 3.1. GRN and Alliin Supplement and Particpants

The BroccoMax/GRN supplements (530 mg) contained 97.7 ± 6.70 µmol g^−1^ glucoraphanin. The Kwai/alliin supplements (715 mg) contained four garlic-derived metabolites: alliin (35.2 ± 0.52 µmol g^−1^), γ-SAC (19.3 ± 1.91 µmol g^−1^), SAC (1.8 ± 0.16 µmol g^−1^), and allicin (21.4 ± 2.10 µmol g^−1^).

**Figure 2:**
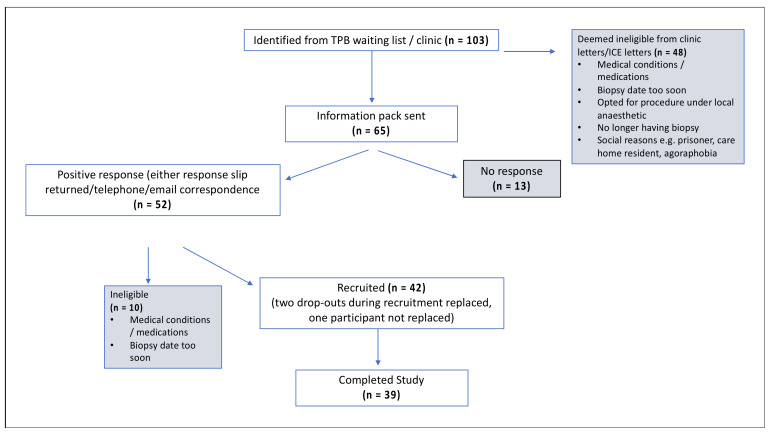
CONSORT diagram with the participant route through the study.

In total, 103 men were identified from the TPB waiting list, of which 65 were deemed eligible and sent a study information pack, and 42 were recruited to the study and randomised to the four arms. A total of 40 participants returned a 24-h urine collection from day one of the study. Participants collected all urine passed following the consumption of four capsules on the morning of day one up until the time of consuming the next day’s capsules. A total of 39 participants underwent TPB of the prostate on the final day (Figure 2, Table 1). All participants who completed the study also completed the CVFFQ and the non-validated allium FFQ on either the day of their biopsy procedure or shortly after. 

There was high inter-individual variation in cruciferous consumption but no statistically significant difference between the intervention groups (Table 2). The non-validated allium questionnaire indicated that there were no outliers in allium consumption amongst the participants and no variation between the study arms The frequency of GSTM1 genotypes was similar to that reported in the general population (Table 2). Histological analysis undertaken on a single tissue core from each participant indicated that tumour was present in 14 of the 39 participants (Table 2).

### 3.2. GRN and Alliin Occurrence in Prostate following Intervention

**Figure 3:**
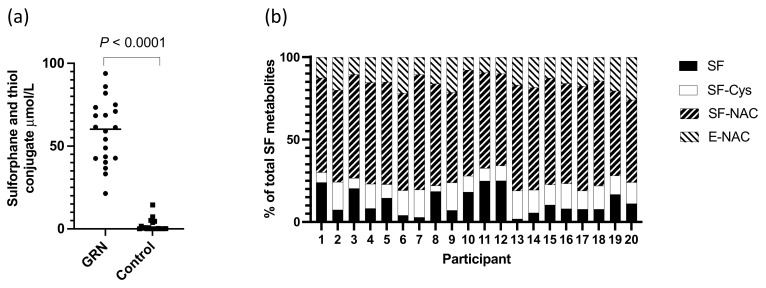
(**a**) Total level of sulforaphane and thiol conjugates in urine of individuals consuming the GRN supplements or not (control); *p*-value from a Mann–Whitney test; (**b**) proportion of sulforaphane and thiol conjugates in urine of participants consuming GRN supplement.

There were significantly higher levels (*p* < 0.0001) of total sulforaphane and its thiol conjugates in urine from participants consuming the GRN-containing intervention compared to the non-GRN containing interventions (Figure 3a). The compounds detected in urine included free sulforaphane and the thiol conjugate sulforaphane-cysteine, sulforaphane-NAC, and erucin-NAC, of which sulforaphane-NAC was the most abundant (Figure 3b). Although the total amount of urinary metabolites detected in urine differed amongst individuals (21.37–93.85 µmol), the proportion of individual metabolites were similar (Figure 3b). The mean excretion of sulforaphane and its metabolites as a percentage of ingested glucoraphanin was 56.21% (range 21–91%, SD ± 18.66). 

**Figure 4:**
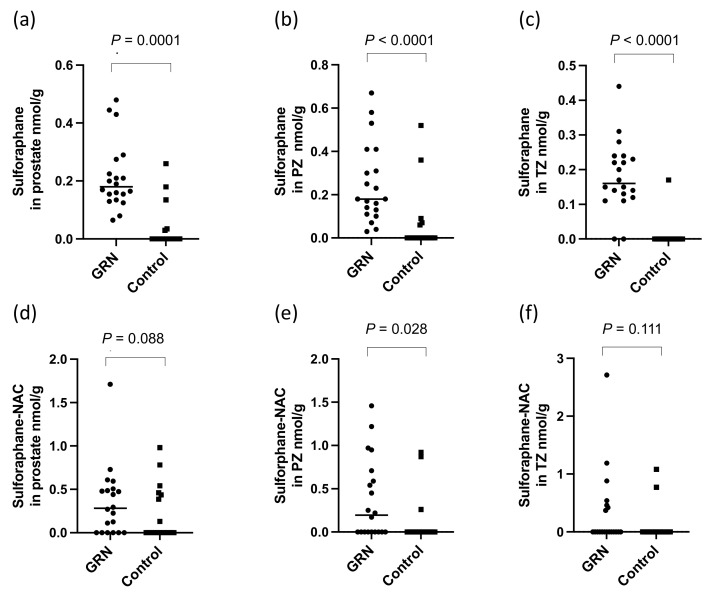
Sulforaphane in (**a**) prostate, (**b**) PZ and (**c**) TZ and sulforaphane-NAC in (**d**) prostate, and (**e**) PZ and (**f**) TZ in prostate following consumption of the GRN supplement or not (control). All *p*-values are from Mann–Whitney tests.

**Figure 5:**
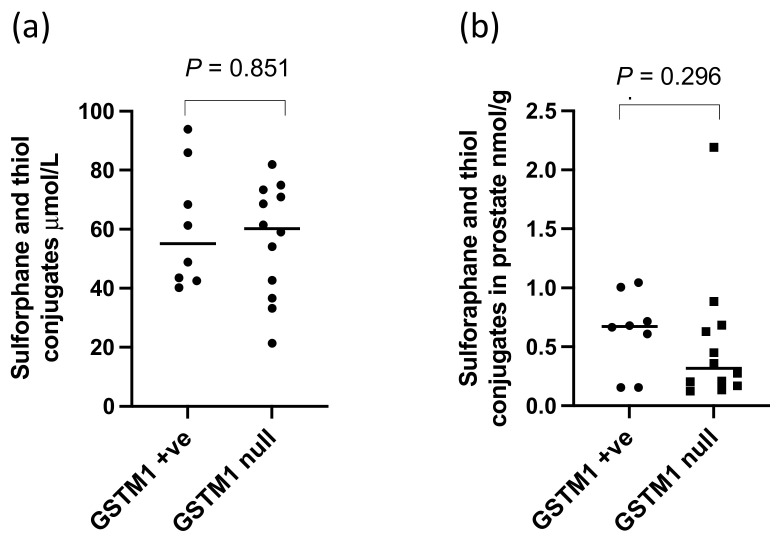
Sulforaphane and thiol conjugates in (**a**) urine and (**b**) prostate of GSTM1 +ve and null participants following GRN supplementation. *p*-values are from Mann–Whitney tests.

There were significantly higher levels of sulforaphane detected in the prostate (*p* < 0.0001, Figure 4a) and within both the PZ and TZ (*p* < 0.0001, Figure 4b,c) in participants receiving the GRN supplements compared to those that did not. The sulforaphane level in the prostate were similar regardless of whether the GRN supplement had been consumed with the alliin supplement or with the placebo supplement (*p* = 0.87), indicative of no interaction between the two supplements. Despite sulforaphane-NAC being the most abundant metabolite in urine (Figure 3b), there was no significant difference in the concentration of sulforaphane-NAC in the prostate between the GRN intervention compared to non-GRN intervention (Figure 4d) although there was an indication that higher concentrations may have occurred in the PZ zone (*p* = 0.028) (Figure 4e,f). Other sulforaphane metabolites were either not detected (SFN-cysteine-glycine and SFN-glutathione) or were below the limits of quantification (SFN-nitrile and erucin-NAC). There was no interaction between the levels of sulforaphane or its metabolites in urine and prostate tissue with GSTM1 genotype (Figure 5a,b). 

**Figure 6:**
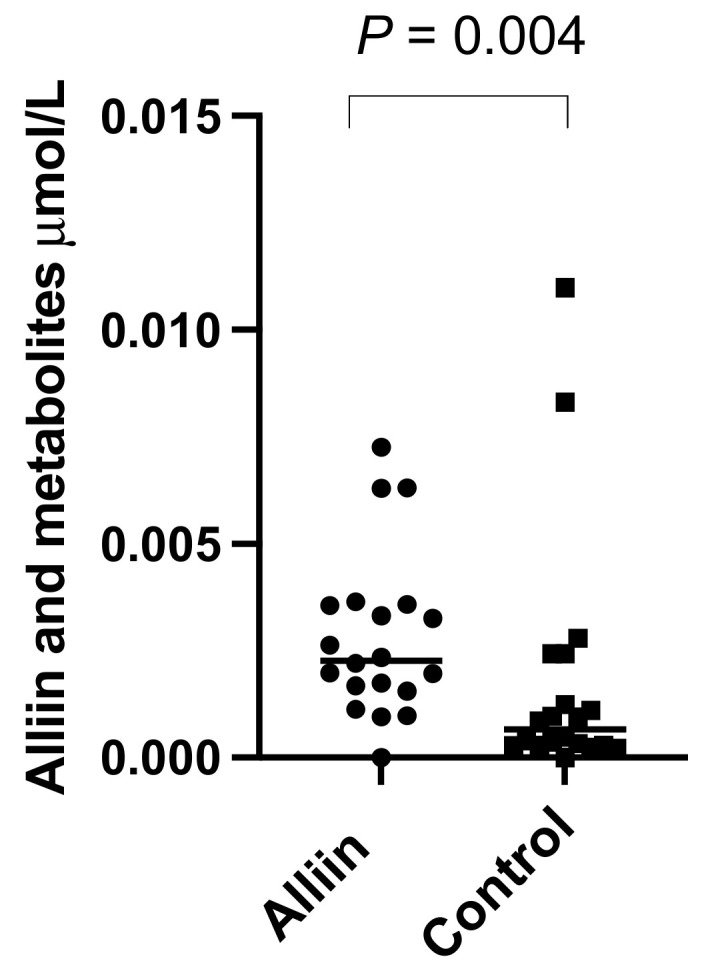
Alliin and associated metabolites in urine of participants consuming alliin supplements or not (control). *p*-values are from Mann–Whitney test.

**Figure 7:**
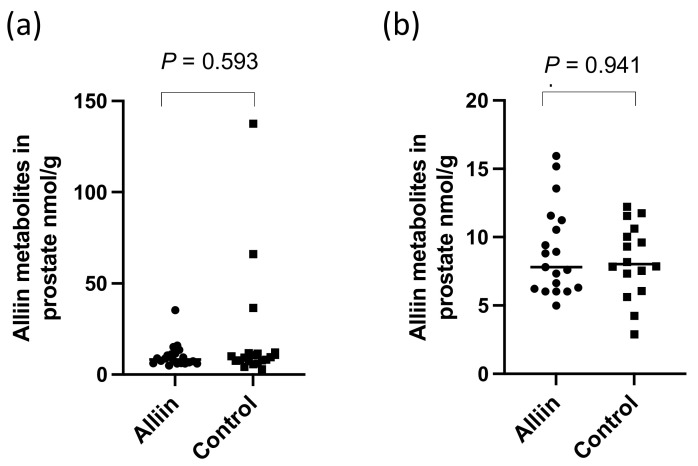
Alliin and associated metabolites in (**a**) prostate tissue and (**b**) with outliers removed. *p*-values are from Mann–Whitney tests.

**Figure 8:**
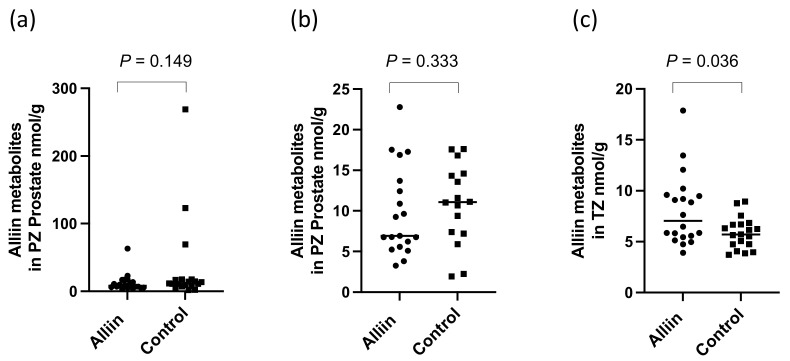
Alliin and associated metabolites in (**a**) PZ, (**b**) PZ with outliers removed, and (**c**) TZ. *p*-values are from Mann–Whitney tests in prostate following consumption of alliin supplements or not (control).

**Figure 9:**
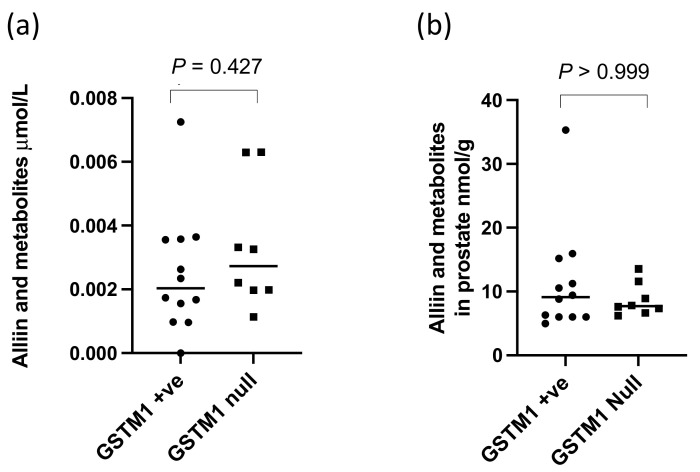
Alliin in metabolites in (**a**) urine and (**b**) prostate of GSTM1 +ve and null participants who consumed the alliin supplement. *p*-values are from Mann–Whitney tests.

There were significantly higher levels of alliin and associated metabolites in the urine from participants who consumed the alliin supplements compared to those who did not (*p* = 0.004) (Figure 6). However, the consumption of the alliin supplements did not affect the concentration of alliin and associated metabolites in the prostate (Figure 7a), which was the case even after the exclusion of several outlier values (Figure 7b). There was some evidence for a greater amount of alliin metabolites in the TZ of men consuming the alliin supplements compared to those who did not (*p* = 0.036) but not for the PZ (Figure 8). There was no association with alliin levels in urine and prostate tissue with GSTM1 genotype (Figure 9a,b). 

## 4. Discussion

There is evidence that certain dietary patterns, such as a Mediterranean diet, and individual dietary components, such as tomatoes, cruciferous vegetables and garlic, may reduce the risk of the incidence or progression of prostate cancer. Certain plant chemicals within these diets and foods have been implicated in mediating protective effects largely on the basis of studies with cell and animal models. Foremost amongst these dietary compounds are lycopene and flavonoids from tomatoes, glucosinolate degradation products including isothiocyanates and indoles from cruciferous vegetables, and alliin and other organo-sulphur compounds from garlic [16]. It is, however, unexplained how these foods and their chemical compounds could influence the biology of the prostate to such an extent that they could reduce carcinogenesis. There are potentially three routes: first, it may be that these foods exert their effects on prostate health indirectly through alterations in hepatic metabolism and improved overall health. Second, it may be that the levels of these compounds in the systematic circulation are at sufficient levels and for a sufficient time to induce changes in prostate biology prior to urinary excretion. In general, however, the levels of dietary phytochemicals and their human metabolites quantified in the circulation are much lower than that used in animal and cell model systems and are very transient, often being undetectable after 24 h. Third, it is conceivable that certain dietary phytochemicals may accumulate in prostate tissue so that there is sufficiently high local concentration to inhibit, for example, the expansion of cancerous cell clones, although to date, this has not been reported. Accumulation could either be directly from the systemic circulation or via urinary reflux, with relatively higher concentrations of dietary metabolites being found in urine than in the blood stream.

In this study, we tested the hypothesis that sulforaphane from the hydrolysis of glucoraphanin from broccoli and alliin from garlic accumulate in the human prostate gland. A factorial design was chosen for the study to improve efficiency, allowing both interventions to be tested independently in the same patient sample. We did not expect any interaction between the two treatments, and there was no evidence from our results that the accumulation of either compound in the prostate was affected by administration of the other.

The urinary excretion of sulforaphane and its metabolites by participants who consumed the GRN supplement was similar to that which has been previously described following the consumption of a broccoli soup [11]. Consistent with previous studies [11,30], there was a variable level of conversion of glucoraphanin to sulforaphane amongst the participants although the ratio of unconjugated sulforaphane to its thiol conjugates in urine was similar amongst participants (Figure 3b) and to that previously reported [30]. The most abundant GRN-derived metabolite in urine was sulforaphane-N-acetyl cysteine. There was little or no sulforaphane or its thiol conjugates detected in the urine of participants who had not consumed the GRN supplement, indicating that they had not consumed broccoli (the major dietary source of glucoraphanin) for at least 24 h prior to sample collection.

The GRN supplement significantly increased the concentration of sulforaphane within the prostate gland (*p* < 0.0001). Some sulforaphane and sulforaphane-N-acetyl cysteine were, however, detected in the prostate glands of men who had consumed the placebo supplement. The participants in the study consumed an average of one portion of cruciferous vegetables per week during the study period, and it is possible that both the sulforaphane and sulforaphane-NAC that occurred within the placebo group was derived from habitual vegetable consumption during the study period. The concentration of sulforaphane excreted in urine or detected in prostate was not influenced by GSTM1 genotype (Figure 5a,b).

Sulforaphane was found in similar amounts within both the peripheral and transition zones of the prostate of the participants who had consumed the GRN supplement. If the origin of sulforaphane in prostate tissue was from urinary reflux, it may be expected that higher levels would occur in the PZ compared to the TZ due to the oblique angle of the ducts emptying from this zone into the prostatic urethra [31]. However, the levels in the PZ and TZ were similar, suggesting the sulforaphane may have been from the systemic circulation. In contrast to sulforaphane, while there was no detectable effect on the concentration of sulforaphane-N-acetyl cysteine in the prostate gland of men who had received the GRN supplement compared to the placebo control, although there was some evidence that it was marginally higher in the PZ (*p* = 0.03, Figure 4e).

It has previously been shown that sulforaphane and its thiol conjugates (measured as dithiocarbamates) are found within human breast tissue following consumption of a broccoli sprout formulation designed to deliver 200 μmol of sulforaphane in contrast to 195 μmol glucoraphanin, as in the present study [32]. It is noteworthy that while in these two studies, similar concentrations of sulforaphane were found in breast and prostate tissue (≈1.7 nmol/g and ≈0.7 nmol/g, respectively), the breast tissue was sampled 1.5 h following consumption of a single broccoli sprout formulation [32], while in the current study, prostate tissue was sampled more than 24 h following consumption of the glucoraphanin supplement that had been provided daily for at least four weeks. 

There is no indication that the presence of sulforaphane within prostate tissue of men who had early-stage prostate cancer would be of concern. One mode of action of sulforaphane is to induce the nrf2 transcription factor that would result in a reduction in oxidative stress and have consequences for gene expression and metabolism that would be likely to reduce the risk of cancer progression. It should be noted that at late stages of tumour growth, enhanced expression of nrf2 may, however, be undesirable [9].

There was a significant difference in the urinary excretion of alliin and associated metabolites in participants who consumed the alliin supplement compared to participants who consumed the placebo or GRN supplement. However, there was no difference in the amount of alliin and associated metabolites in the prostate gland of men who had consumed the alliin supplement compared to placebo and GRN supplements although it was marginally higher in the TZ (*p* = 0.036, Figure 8c). Similar to sulforaphane, there was no interaction between levels of alliin and GSTM1 genotype (Figure 9a,b). It is noteworthy that alliin was detected within the prostate of every participant and that a small number of prostate tissue samples within both arms of the study had much higher concentrations of alliin than the majority of samples. Removal of these outliers made no difference to the statistical analyses. The estimation of dietary intake of alliaceous vegetables is challenging due to their widespread presence in processed foods, and it is likely that intake is often underestimated. Additional studies are required to further understand the metabolism, tissue accumulation, and excretion of alliin. It is conceivable that alliin does accumulate in human prostate tissue, but its turnover is much slower than that of sulforaphane so that a longer “allium-free diet” is required prior to an intervention to assess its accumulation. 

To conclude, we provide evidence that sulforaphane can be detected in human prostate tissue following regular consumption of glucoraphanin supplements. In contrast, alliin and associated metabolites were not more abundant in the prostates of men receiving the alliin garlic-derived supplement (with the exception of possibly higher levels specifically in the TZ) but were present in all samples analysed. This may suggest that the dynamics of alliin accumulation, metabolism, and elimination from the prostate contrast that of sulforaphane. 

If dietary metabolites were to enter the prostate through urinary reflux, it may be expected that there would be higher levels in the PZ compared to the TZ. This is not the case for either sulforaphane or alliin. The prostate gland has a complex arterial network via the prostatic arteries which are mainly derived from the internal iliac arteries, with other branches arising from the middle rectal and pudendal arteries. The fact that up to 60% of men demonstrate considerable anastomoses between prostatic pedicles and surrounding arteries makes exposure to the prostate through the systemic circulation a plausible hypothesis [33]. 

The accumulation of sulforaphane and presence of alliin in prostate tissue, as demonstrated in this study, may result in local effects on healthy and cancerous cells through a variety of mechanisms, which may explain the reduced risk of prostate cancer incidence and progression following consumption of cruciferous and alliaceous vegetables.

## Figures and Tables

**Table 1:** Inclusion and exclusion criteria.

Basic Inclusion Criteria	Basic Exclusion Criteria
Males	Those regularly taking 5α-reductase inhibitors or testosterone replacement medicines
Aged 18–80 years	Those on warfarin treatment
BMI between 19.5–35 kg/m^2^	Those diagnosed with diabetes
Smokers and non-smokers	Those diagnosed with or suspected to be high-risk for human immunodeficiency virus (HIV) and/or viral hepatitis
Scheduled for TPB as part of routine investigation or staging of prostate cancer	Those allergic to any of the ingredients included in the supplements
	Those taking additional dietary supplements or herbal remedies that could affect the study outcome
	Those that are unable to understand English or give informed consent
	Parallel participation in another research project that involves dietary intervention
	Any person related to or living with any member of the study team

**Table 2:** Participant demographics, cruciferous vegetable consumption, and histology. The non-validated allium questionnaire did not indicate any outliers in allium consumption amongst the participants.

	Broccomax/Placebo	Broccomax/Kwai	Kwai/Placebo	Placebo/Placebo
**Number of participants**	10	10	10	9
**Age (years)**	67.4 ± 5.62	64.3 ± 8.15	63.5 ± 5.70	63.8 ± 5.29
**BMI (kg/m^2^)**	26.5 ± 2.94	26.7 ± 2.89	26.0 ± 4.86	27.2 ± 5.00
**Cruciferous Vegetable Intake (servings/day)**	1.1 ± 0.66	1.1 ± 0.55	1.0 ± 0.37	0.6 ± 0.53
**GSTM1 (Null, Heterozygous, homozygous)**	7, 3, 0	5, 4, 1	5, 2, 3	7, 3, 0
**Histology (Benign, malignant)**	6, 4	5, 5	7, 3	7, 2

## Data Availability

Data are available upon request for the corresponding author.
